# Effects of orthokeratology lenses on choroidal thickness and choriocapillaris perfusion in myopic children

**DOI:** 10.3389/fmed.2025.1620042

**Published:** 2025-08-05

**Authors:** Lulu Wang, Yu Liu, Mengyao Ma, Yifan Zhao, Xiuli Bao

**Affiliations:** ^1^Department of Ophthalmology, The Affiliated Hospital of Inner Mongolia Medical University, Hohhot, China; ^2^School of Public Health, Inner Mongolia Medical University, Hohhot, China

**Keywords:** children, myopia, orthokeratology, choroid, choriocapillaris, OCT

## Abstract

**Purpose:**

To investigate changes in subfoveal choroidal thickness (SFChT) and choriocapillaris (CC) perfusion in children wearing orthokeratology (Ortho-K) lenses.

**Methods:**

This retrospective study included 61 myopic children, who were divided into two groups. The Ortho-K group wore Ortho-K lenses, while the control group wore single-vision distance spectacles. The SFChT, CC area and ocular parameters were measured before and after 1 week, 1 month, 3 months, and 6 months of treatment.

**Results:**

In the Ortho-K group, the SFChT significantly increased by 18.23 ± 11.35 μm. The CC area significantly increased by 0.39 ± 0.11 mm^2^ from baseline at the 1-week visit and continued to increase at the 1-month, 3-month, and 6-month follow-ups. In the control group, the choroidal parameters did not change significantly at 1 week. At the 1-, 3-, and 6-month follow-ups, the control group showed a notable reduction in SFChT and CC areas (*p* < 0.001). At the 6-month follow-up, there was a significant relationship between changes in SFChT and CC areas in the Ortho-K group (*r* = 0.890, *p* < 0.001). The increasing SFChT and CC areas were negatively correlated with AL (*r* = −0.827, *p* < 0.001; *r* = −0.717, *p* < 0.001), weakly negatively correlated with changes in VCD (*r* = −0.033, *p* < 0.05; *r* = −0.039, *p* < 0.05), and negatively correlated with K1 and K2 (*r* = −0.430, *p* < 0.001 and *r* = −0.545, *p* < 0.001, respectively; *r* = −0.417, *p* < 0.001 and *r* = −0.464, *p* < 0.001, respectively).

**Conclusion:**

Orthokeratology notably increases SFChT and improves CC perfusion. Changes in choroidal thickness are linked to alterations in choriocapillaris perfusion. These effects are associated with the effect of orthokeratology in slowing axial length elongation.

## Introduction

Myopia is a common eye condition in East Asia, notably in China, affecting children and adolescents. Its prevalence is increasing each year (1, 2). If myopia develops early, it progresses more quickly, raising the chances of high myopia, which significantly increases the risk of eye conditions such as retinal detachment, myopic maculopathy, and glaucoma (3, 4). Thus, addressing myopia in children and adolescents is now a major public health issue.

In the past decade, orthokeratology lenses have been extensively utilized for myopia control in Chinese children and teenagers and are regarded as the most effective method for slowing myopia progression (5, 6). The orthokeratology process involves wearing a specially designed gas-permeable lens overnight, which alters the shape of the cornea to induce peripheral myopic defocus and slow the growth of axial length (7). Nonetheless, the exact mechanisms through which it acts are still not completely understood.

Recent studies have shown that changes in the choroid’s structure and blood flow perfusion may play a key role in myopia progression (8). Both drug treatments and optical interventions for myopia can impact the choroid (9, 10). The precise role of the choroid in the optical treatment of myopia with orthokeratology lenses in children and adolescents is still unclear.

Optical Coherence Tomography Angiography (OCTA) is a non-invasive approach that has seen widespread use recently, attributed to its deep scanning abilities and quantitative assessment of retinal and choroidal vascular structures. Certain clinical studies have indicated that orthokeratology may lead to an increase in subfoveal choroidal thickness (SFChT) (11, 12). While these studies mainly address the overall choroidal blood flow perfusion or changes in the larger vascular layer beneath the fovea (13), few focus on the choriocapillaris layer.

This retrospective study aims to evaluate the effects and determinants of orthokeratology lens intervention on myopia. We employed optical coherence tomography (OCT) and OCTA to examine alterations in choroidal thickness and choriocapillaris perfusion beneath the fovea—a critical area for visual function in myopic children following orthokeratology lens usage. The findings offer novel insights into the mechanisms underlying myopia control strategies for clinical research.

## Methods

This retrospective study was conducted in the Department of Ophthalmology at the Affiliated Hospital of Inner Mongolia Medical University between June 2022 and June 2024. The study received approval from the Ethics Committee of Inner Mongolia Medical University (Hohhot, China) and was conducted in strict accordance with the principles outlined in the Declaration of Helsinki (Approval No. YKD202202003). Written informed consent was obtained from all participants and their parents or guardians.

### Inclusion and exclusion criteria

The study included children diagnosed with myopia at the affiliated hospital who underwent either orthokeratology or single-vision lens correction.

The inclusion criteria were as follows: (1) Residency in urban Hohhot, China; (2) age between 8 and 14 years; (3) binocular cycloplegic spherical equivalent (SE) ranging from −5.00 to −1.00 diopters (D) post-cycloplegia; (4) best-corrected visual acuity (BCVA) of 20/20 or better; (5) astigmatism of ≤1.00 diopters cylinder (DC). (6) Complete follow-up data available includes corneal topography, ocular biometry, and OCT and OCTA examinations of the fundus at the start and after 6 months of daily Ortho-K lens wear.

The exclusion criteria were: (1) presence of strabismus and binocular vision abnormalities, including anomalies in binocular fusion and defects in stereoscopic vision; and (2) any history of ophthalmic or systemic diseases and abnormalities; (3) Prior utilization of various myopia control strategies, including progressive addition lenses, defocus incorporated multifocal (DIMS) spectacles, contact lenses, and low-concentration atropine medication, was noted.

### Examination

In the examination phase, all participants underwent cycloplegic refraction using 1% atropine prior to enrollment to diagnose newly developed myopia. Comprehensive ophthalmic evaluations were performed before initiating treatment, which included the assessment of best-corrected visual acuity (BCVA). Given the potential impact of cycloplegics on the choroid, the study collected non-cycloplegic automated refraction data (Nidek ARK-510A, Nidek Inc., Tokyo, Japan) and calculated the spherical equivalent refraction (SER) as the sphere power plus half of the cylinder power. Ocular biometry (IOLMaster700, Carl Zeiss, Germany) was utilized to measure axial length (AL), anterior chamber depth (ACD), lens thickness (LT), and white-to-white distance (WTW), with vitreous depth calculated as AL minus the sum of central corneal thickness (CCT), ACD, and LT. Scheimpflug corneal tomography (Pentacam, Oculus GmbH, Wetzlar, Germany) was employed to obtain K1 (flat keratometry), K2 (steep keratometry), and corneal astigmatism measurements. Additionally, slit-lamp examination and direct ophthalmoscopy were conducted. All measurements were conducted between 14:30 and 18:00 to minimize the impact of diurnal variations on choroidal parameter assessments.

### Intervention measures

Participants in the Ortho-K group were fitted with Alpha ortho-k VST lenses (ALPHA, Nagoya, Japan) for both eyes. They were instructed to wear these lenses during nighttime sleep for a minimum of 8 continuous hours daily. In contrast, the control group underwent cycloplegic refraction followed by the fitting of single-vision spectacles.

### Follow-up

Assessments were conducted prior to treatment and subsequently at 1 week, 1 month, 3 months, and 6 months post-treatment. During each follow-up visit, participants underwent a slit-lamp examination to identify any contraindications for orthokeratology lens use, such as severe corneal injury, allergies, or infections. Cases presenting with such conditions were excluded from the study.

### Choroidal parameter measurements

We employed the RTVue XR OCT spectral domain optical coherence tomography (SD-OCT) system, integrated with AngioVue version equipment (Optovue, Fremont, CA, United States), which operates at a high velocity of 70,000 scans per second at a wavelength of 840 nm. This system provides an axial resolution of 5 μm and a lateral resolution of 15 μm. OCT angiography (OCTA, ReVue, Optovue, Inc., Fremont, CA) was utilized to perform a 6 × 6 mm^2^ scan centered on the macula. SFChT was measured using SD-OCT instrumentation to determine the distance from the retinal pigment epithelium to the posterior edge of the scleral junction. The CC layer was automatically segmented from 10 μm above to 30 μm below Bruch’s membrane (BM). The software generated an en face slab image of the CC and automatically computed the blood flow area values for the CC layer within the specified 6 × 6 mm scan area. All OCT and OCTA images were captured by the same experienced operator, and only images with a quality index exceeding 80 were selected for analysis. Image processing was independently conducted by two trained graduate students, and the mean value of their measurements was used for further analysis.

### Statistical analysis

Data analysis was conducted using SPSS version 27.0. Initially, the normality of all quantitative data was evaluated using the Shapiro–Wilk test. Data following a normal distribution were presented as $\bar{x}\pm s$ , and intergroup differences were evaluated using t-tests. For within-group analyses, repeated measures ANOVA was conducted to assess variations across different time points, followed by pairwise comparisons using the Least Significant Difference (LSD) *post hoc* test. Data that were not normally distributed were represented by the median and interquartile range, with intergroup differences assessed via the Wilcoxon rank-sum test. Categorical data were showed as frequencies or percentages, and with intergroup comparisons performed using the chi-square (χ^2^) test or Fisher’s exact test. Ordinal data were similarly expressed as frequencies or percentages, with intergroup differences evaluated using the Wilcoxon rank-sum test. Spearman’s rank correlation coefficient was employed to analyze correlations between variables, including SFChT and changes in CC area. A significance threshold of α = 0.05 was set, and *p*-values below 0.05 were deemed to indicate statistically significant differences.

## Results

From June 2022 to June 2024, a total of 61 myopic children (31 males and 30 females) met the inclusion criteria for this study. The Ortho-K group included 30 cases, and the control group included 31. Only data from the right eye were used for further analysis in this study. A comprehensive summary of the baseline characteristics for both the Ortho-K group and the control group is presented in Table 1. Initially, no statistically significant differences were observed between the two groups.

**Table 1:** Baseline characteristics in the two study groups.

Participant characteristics	Ortho-K (*N* = 30)	Control (*N* = 31)	*t/χ* ^2^	*P*-value
Age	10.3 ± 2.37	10.61 ± 2.01	0.556	0.581
Gender (F/M)	16/14	14/17	0.146	0.702
AL (mm)	24.39 ± 0.64	24.15 ± 0.9	−1.244	0.219
SER (D)	−2.64 ± 1.19	−2.73 ± 1.2	−0.301	0.764
ACD (mm)	3.66 ± 0.23	3.65 ± 0.23	−0.253	0.801
WTW (mm)	12.24 ± 0.46	12.3 ± 0.58	0.447	0.657
K1 (D)	42.74 ± 1.55	42.75 ± 1.41	0.024	0.981
K2 (D)	43.41 ± 1.51	43.38 ± 1.4	−0.086	0.932
VCD (mm)	16.85 ± 0.78	16.61 ± 1.06	−1.002	0.321
SFChT (μm)	25.60 ± 0.87	25.56 ± 1.18	−0.149	0.882
CC aeras (mm^2^)	310.20 ± 63.62	311.52 ± 67.01	0.079	0.938

AL, axial length; SER, spherical equivalent refraction; ACD, anterior chamber depth; WTW, white-to-white; K1, flat keratometry; K2, steep keratometry; VCD, vitreous chamber depth; SFChT, subfoveal choroidal thickness; CC, choriocapillaris.

In the Ortho-K group, AL and VCD showed slight reductions from baseline at the 1-week and 1-month follow-ups, while at the 3-month and 6-month follow-ups, AL and VCD showed slight increases from baseline (repeated measures ANOVA, AL: *F* = 9.398, *p* < 0.001; VCD: *F* = 3.421, *p* = 0.041, respectively). In the control group, AL and VCD exhibited slight increases from baseline at the 1-week and 1-month follow-ups, and significant increases from baseline at the 3-month and 6-month follow-ups (repeated measures ANOVA, AL: *F* = 13.394, *p* < 0.001; VCD: *F* = 35.418, *p* < 0.001, respectively). Significant differences were observed in AL and VCD measurements between the two groups (repeated measures ANOVA, AL: *F* = 4.014, *p* = 0.019; VCD: *F* = 5.541, *p* < 0.001, respectively).

In the Ortho-K group, significant changes in SER, K1, and K2 from baseline were observed at the 1-week follow-up, and these differences persisted until the 6-month follow-up (repeated measures ANOVA, SER: *F* = 30.746, *p* < 0.001). Meanwhile, no significant changes in K1 and K2 were detected throughout the follow-up period (repeated measures ANOVA, K1: *F* = 0.842, *p* = 0.395; K2: *F* = 0.718, *p* = 0.485, respectively). Significant differences were observed in the measurements of SER, K1, and K2 between the two groups (repeated measures ANOVA, SER: *F* = 8.647, *p* = 0.001; K1: *F* = 11.001, *p* < 0.001; K2: *F* = 10.265, p < 0.001, respectively).

No significant differences were observed in ACD and WTW measurements between the two groups (repeated measures ANOVA, ACD: *F* = 0.444, *p* = 0.687; WTW: *F* = 0.123, *p* = 0.828, respectively) (Table 2).

**Table 2:** Comparison of changes from baseline in ocular parameters between the Ortho-K group and the control group during a 6-month follow-up period.

Parameters	Ortho-K (*N* = 30)	Control (*N* = 31)	*t*	*P*
AL (mm)				
1-week	−0.03 ± 0.09	0.01 ± 0.04	2.387	0.022
1-month	−0.01 ± 0.07	0.07 ± 0.13	−2.833	0.006
3-month	0.03 ± 0.07	0.15 ± 0.20	5.996	<0.001
6-month	0.07 ± 0.10	0.21 ± 0.25	2.748	0.009
SER (D)				
1-week	1.90 ± 0.45	−0.04 ± 0.08	−23.549	<0.001
1-month	2.32 ± 1.14	−0.13 ± 0.08	−11.770	<0.001
3-month	2.32 ± 0.57	−0.24 ± 0.14	−23.838	<0.001
6-month	2.45 ± 0.61	−0.42 ± 0.23	−23.897	<0.001
ACD (mm)				
1-week	−0.01 ± 0.03	0 ± 0.03	1.828	0.073
1-month	0 ± 0.07	0 ± 0.06	0.318	0.752
3-month	0.01 ± 0.04	0.01 ± 0.04	0.074	0.941
6-month	0.01 ± 0.04	0.01 ± 0.04	−0.218	0.828
WTW (mm)				
1-week	0.04 ± 0.11	0.05 ± 0.15	−0.259	0.796
1-month	0.05 ± 0.16	0.02 ± 0.2	−0.654	0.516
3-month	0.09 ± 0.28	0.07 ± 0.22	−0.341	0.734
6-month	0.15 ± 0.44	0.14 ± 0.34	−0.111	0.912
K1 (D)				
1-week	−1.13 ± 0.66	−0.02 ± 0.1	9.172	<0.001
1-month	−1.42 ± 0.76	0.02 ± 0.21	9.965	<0.001
3-month	−1.61 ± 0.79	0.08 ± 0.29	11.021	<0.001
6-month	−2.19 ± 0.96	0.06 ± 0.56	11.11	<0.001
K2 (D)				
1-week	−1.35 ± 0.51	0.01 ± 0.09	14.492	<0.001
1-month	−1.66 ± 0.45	−0.01 ± 0.31	16.627	<0.001
3-month	−2.00 ± 0.63	0.02 ± 0.41	14.876	<0.001
6-month	−2.26 ± 1.04	0.09 ± 0.52	11.083	<0.001
VCD (mm)				
1-week	−0.10 ± 0.01	0.00 ± 0.02	22.867	<0.001
1-month	−0.05 ± 0.03	0.02 ± 0.05	6.383	<0.001
3-month	0.02 ± 0.07	0.05 ± 0.03	2.479	0.017
6-month	0.01 ± 0.08	0.14 ± 0.15	4.175	<0.001

**Figure 1:**
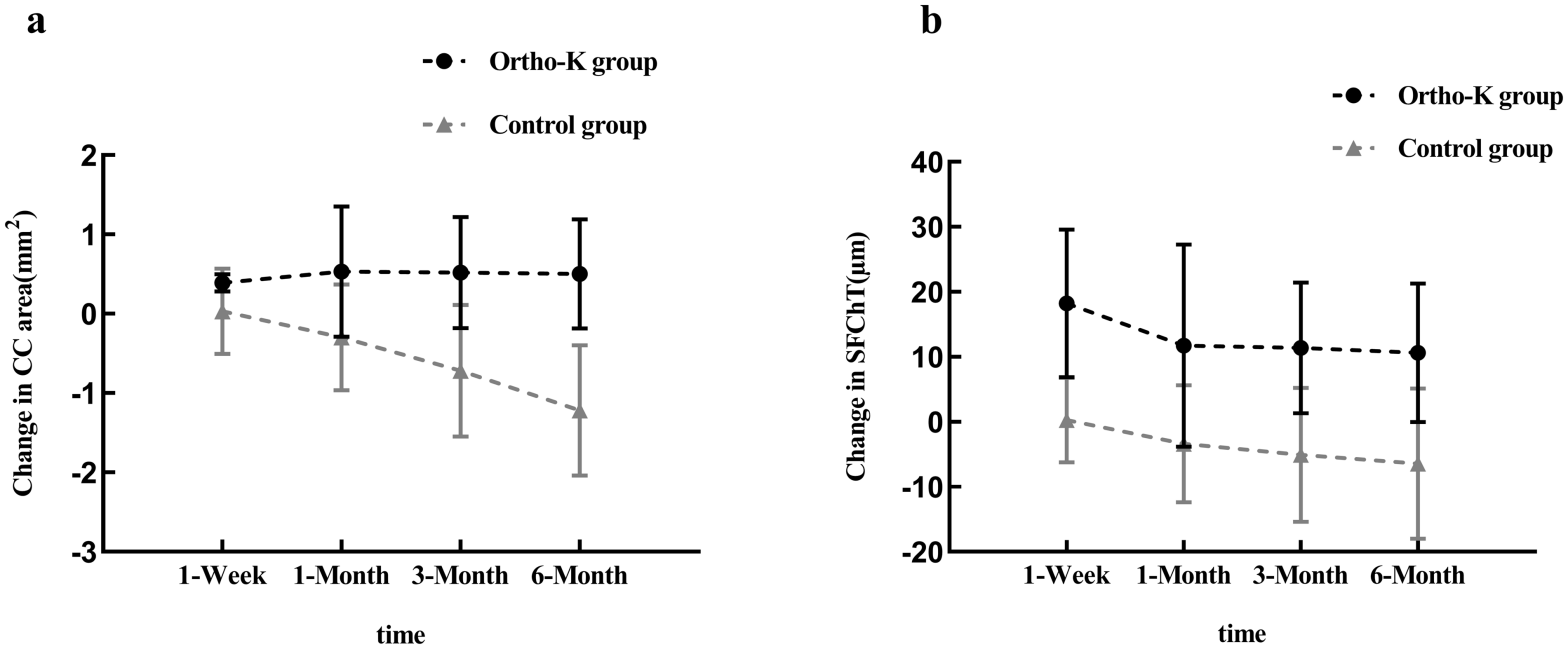
Changes in CC areas and SFChT over time for both groups. Line charts showing the changes in **(a)** CC areas and **(b)** SFChT over the 6-month follow-up period between the two groups.

During the 6-month follow-up period, alterations in choroidal parameters were statistically significant in both the Ortho-K and control groups. In the Ortho-K group, both the CC area and the SFChT showed a significant increase compared to baseline at the 1-week follow-up, with this increase persisting through the 6-month follow-up (Figure 1; Tables 3, 4). During the early follow-up period, within the first week and the first month, the the CC area showed a significant increase from baseline. This increasing trend persisted throughout the 6-month follow-up (*F* = 0.330, *p* > 0.05) (Figure 1a; Table 3). Similarly, the most pronounced increase in SFChT was observed during the first week of follow-up. Although the thickening of SFChT decreased at the 1-month, 3-month, and 6-month follow-ups compared to the first week, it remained significantly higher than baseline (*F* = 4.002, *p* < 0.05) (Figure 1b; Table 4). Conversely, in the control group, no significant changes were observed in the CC area and SFChT at the 1-week follow-up. However, the control group demonstrated a reduction in both the CC area and SFChT at the 1-month follow-up, with this trend continuing through the 3-month and 6-month follow-ups (CC area: *F* = 37.486, *p* < 0.001; SFChT: *F* = 15.072, *p* < 0.001, respectively) (Figure 1; Tables 3, 4). At each follow-up time point, significant differences were observed between the Ortho-K group and the control group in terms of SFChT and CC area (CC area: *F* = 16.733, *p* < 0.001; SFChT: *F* = 0.388, *p* < 0.001, respectively) (Figure 1; Tables 3, 4).

**Table 3:** Changes in CC area from the baseline during 6-month follow-up period.

Group	1-week	1-month	3-month	6-month	*F*	*P*
Ortho-K (*N* = 30)	0.39 ± 0.11	0.53 ± 0.82	0.52 ± 0.70	0.50 ± 0.69	0.330	0.688
Control (*N* = 31)	0.03 ± 0.54	−0.3 ± 0.67^a^	−0.72 ± 0.83^ab^	−1.22 ± 0.82^abc^	37.486	<0.001
*t*	−3.626	−4.303	−6.290	−8.883		
*P*	<0.001	<0.001	<0.001	<0.001		

^a^*P* < 0.05:vs 1-week, ^b^*P* < 0.05:vs 1-month, ^c^*P* < 0.05:vs 3-month.

Group effect: *F* = 68.778, *P* < 0.001; time effect: *F* = 12.605, *P* < 0.001; time × group: *F* = 16.733, *P*<0.001.

**Table 4:** Changes in SFChT from the baseline during a 6-month follow-up period.

Group	1-week	1-month	3-month	6-month	*F*	*P*
Ortho-K (*N* = 30)	18.23 ± 11.35	11.73 ± 15.55	11.37 ± 10.07^a^	10.60 ± 10.70^a^	4.002	0.024
Control (*N* = 31)	0.29 ± 6.50	−3.39 ± 9.00^a^	−5.10 ± 10.34^ab^	−6.42 ± 11.54^a^	15.072	<0.001
*t*	−7.544	−4.628	−6.301	−5.977		
*P*	<0.001	<0.001	<0.001	<0.001		

^a^*P* < 0.05:vs 1-week, ^b^*P* < 0.05:vs 1-month, ^c^*P* < 0.05:vs 3-month.

Group effect: *F* = 54.690, *P* < 0.001; time effect: *F* = 11.257, *P* < 0.001; time × group: *F* = 0.388, *P* < 0.001.

**Figure 2:**
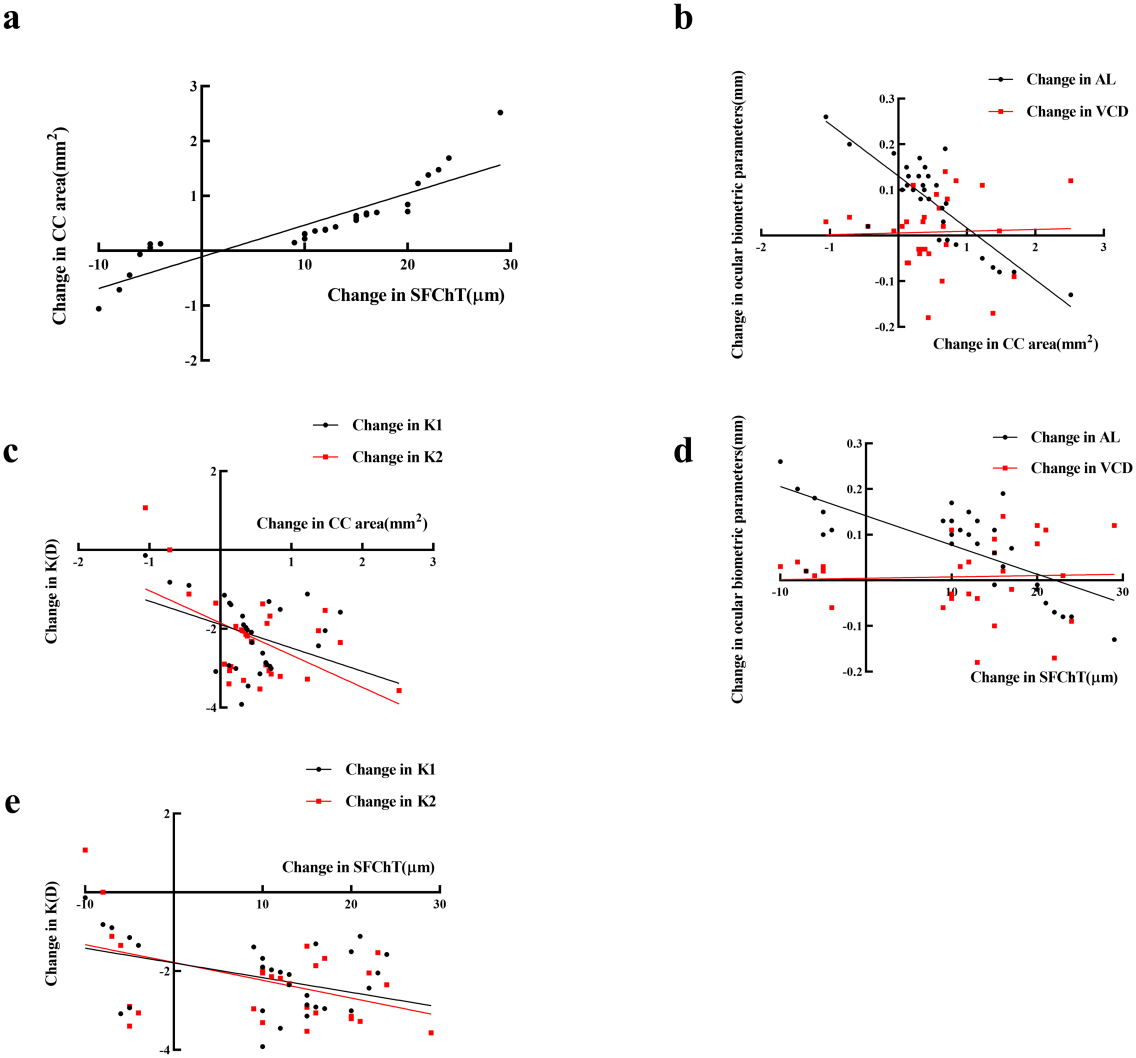
Correlation between changes in choroidal parameters and changes in ocular biometric parameters at the 6-month follow-up in the Ortho-K group. **(a)** Change in SFChT was positively correlated with change in CC area. **(b)** Change in CC area was negatively correlated with AL and weakly negatively correlated with change in VCD. **(c)** Change in CC area was negatively correlated with changes in K1 and K2. **(d)** Change in SFChT was negatively correlated with AL and weakly negatively correlated with change in VCD. **(e)** Change in SFChT was negatively correlated with changes in K1 and K2.

At the 6-month follow-up, the Ortho-K group showed a strong positive correlation between increased CC area and SFChT (*r* = 0.890, *p* < 0.001, Figure 2a). The CC area increase was negatively correlated with AL (*r* = −0.827, *p* < 0.001), weakly negatively with VCD changes (*r* = −0.033, *p* < 0.05, Figure 2b), and negatively with K1 and K2 changes (*r* = −0.430, *p* < 0.001 and *r* = −0.545, *p* < 0.001, Figure 2c) (Figure 2).

SFChT thickening was negatively correlated with AL (*r* = −0.717, *p* < 0.001), weakly negatively with VCD changes (*r* = 0.039, *p* < 0.05, Figure 2d), and negatively with K1 and K2 changes (*r* = −0.417, *p* < 0.001 and *r* = −0.464, *p* < 0.001, Figure 2e) (Table 5).

**Table 5:** Linear regression analysis of choroidal parameters in relation to ocular biometric parameters in the Ortho-K group at 6 months follow-up.

Parameters	CC area	SFChT	AL	K1	K2	VD
CC area	1					
SFChT	0.890^**^	1				
AL	−0.827^**^	−0.717^**^	1			
K1	−0.430^*^	−0.417^*^	0.226	1		
K2	−0.545^**^	−0.464^**^	0.352	0.340	1	
VCD	0.033	0.039	−0.043	−0.006	−0.102	1

*P < 0.05; **P < 0.01.

## Discussion

This retrospective study aimed to investigate the alterations over time in SFChT and choriocapillaris blood perfusion in myopic children undergoing orthokeratology treatment, as well as the local factors influencing these changes. The findings revealed that after orthokeratology intervention, the SFChT significantly increased, an expansion in the area of choriocapillaris perfusion in the macular region, and a positive correlation was observed between changes in SFChT and alterations in choriocapillaris blood flow. These results suggest that the efficacy of orthokeratology in controlling myopic progression is linked to both structural modifications and changes in capillary blood perfusion within the choroid.

Numerous studies consistently demonstrate that orthokeratology effectively mitigates progression of myopia in children by decelerating the growth of ocular AL (14, 15). There is a significant correlation between children’s choroidal thickness (ChT) and age, refractive errors, and AL. Multiple studies have reported a correlation between children’s ChT and AL. The development of children’s ChT exhibits a bimodal pattern with age. On one hand, ChT increases due to the natural growth of the eye during childhood, continuing until puberty. On the other hand, in children with myopia, the thickening of ChT is restricted by the elongation of axial length and the progression of myopia (16). Read et al. proposed that there is a positive correlation between ChT and age in emmetropic children, whereas in myopic children, this relationship is negative (17). Read et al. also found that the direct observation of children’s SFChT was negatively correlated with AL (18), while Jin et al. reported an independent correlation between choroidal thinning and AL (19). Recent investigations have documented that orthokeratology leads to thicker choroids in children with myopia, although the specific regions of choroidal thickening reported have varied. Li et al. (20) conducted a longitudinal study involving 29 children aged 9–14 years who were treated with ortho-K lenses, finding that SFChT increased by 16 μm compared to baseline after 1 month. Similarly, Chen et al. (14) observed an increase in choroidal thickness at the parafoveal region by 21.8 ± 25.2 μm in a cohort of 39 myopic children aged 7 to 17 years following 3 weeks of orthokeratology treatment. Jin et al. (21) utilized Cirrus-HD OCT technology to investigate the effects of orthokeratology on 30 myopic children with a mean age of 11.3 ± 1.7 years. Their research indicated that after 3 months of wearing Ortho-K lenses, there was a marked increase in ChT, especially in the temporal region in the horizontal direction. This region is chiefly influenced during the progression of myopia and is more likely to undergo changes from optical defocus than the nasal region. Our study focused on the central fovea, the area with the highest visual acuity, as the primary site for detection. The findings indicated that myopic children undergoing orthokeratology treatment experienced a significant increase in SFChT within 1 week of lens wear, with this thickening persisting unchanged over a 6-month follow-up period. Additionally, there was a negative correlation between the thickening of SFChT and alterations in AL. However, it remains uncertain whether changes in ChT precede or follow changes in AL.

The mechanism by which orthokeratology slows axial elongation and induces choroidal thickening is primarily attributed to the peripheral myopic defocus created by Ortho-K lenses, which is a recognized method for controlling myopic progression. Peripheral defocus leads to choroidal thickening, which subsequently moves the retina toward the defocus interface to correct refractive errors (13). Animal experiments have demonstrated that alterations in choroidal thickness mediate the impact of defocus on AL (22), and clinical studies have similarly reported rapid bidirectional changes in axial length under specific defocus conditions, likely driven by modifications in choroidal thickness (23). The precise mechanisms underlying the choroidal thickening induced by defocus remain unclear. However, changes in the tone of non-vascular choroidal smooth muscle or variations in choroidal blood flow have been proposed as potential mechanisms for the defocus-induced changes in choroidal thickness (24).

Our study revealed that after 1 week of Ortho-K lens wear, there was a significant increase in the CC area among myopic children. This change is closely associated with an increase in choroidal thickness and a deceleration in axial eye growth. It is hypothesized that, due to the proximity of the CC to the retina, defocus signals originating from the retina may rapidly stimulate an expansion in the CC blood perfusion. This alteration in the CC could represent a critical mechanism through which Ortho-K lenses influence changes in AL and SFChT. In myopia, abnormal variations in choroidal blood flow are frequently accompanied by changes in choroidal thickness. In myopic animal models, modifications in choroidal blood flow have been observed to precede changes in choroidal thickness (25, 26). Previous studies by Gupta et al. have demonstrated that thinning of the choroid in myopia is associated with a reduction in the vascular components and matrix of the choroid (27). Additionally, Liu et al. (28) confirmed a significant decrease in choroidal stroma volume (CSV) and choroidal vessel volume (CVV) in children with myopia. Despite inconsistent reports on the impact of defocus on blood perfusion across different choroidal layers (11, 29), recent studies indicate that Ortho-K lenses may influence choroidal thickness by affecting choroidal blood perfusion in the fovea (12, 30). Our study assessed the effect of the choriocapillaris area as an analytical indicator for myopic control in children using Ortho-K lenses. We observed that an increase in the CC area is associated with effective myopia control, whereas a decrease may correspond to myopic regression. Furthermore, compared to medium-to-large choroidal vessels, the choriocapillaris exhibits more pronounced changes in blood flow in myopic eyes, experiences fewer circadian rhythm fluctuations, and allows for more precise identification and quantification due to reduced roll-off attenuation of OCT signals (10, 31). Consequently, the choriocapillaris may serve as a valuable indicator for monitoring myopia progression and evaluating the efficacy of myopia prevention and control strategies.

The present study is subject to certain limitations. Firstly, the research exclusively assessed choroidal thickness beneath the fovea, rather than evaluating both choroidal thickness and volume across all quadrants of the macula. This limitation underscores the need for further structural investigations encompassing a more extensive area of the choroid. Secondly, the study concentrated on alterations in choroidal blood flow attributable to orthokeratology lenses. However, due to signal attenuation in OCTA and the absence of distinct stratification between medium and large vessels within the choroid, a comprehensive layer-by-layer quantitative analysis of choroidal vessels was not feasible. This limitation highlights the necessity for more sophisticated computational techniques to facilitate detailed future studies. Thirdly, the restricted sample size calls for additional research involving larger cohorts to enhance the robustness of the conclusions. Therefore, future studies should aim to assess the influence of a wider array of choroidal structures and hemodynamic parameters on the effects of orthokeratology lenses.

## Conclusion

Orthokeratology increases SFChT and choriocapillaris perfusion, notably after 1 week and consistently over 6 months. These changes may relate to alterations in AL and could indicate myopia control effectiveness and myopic progression. Further research is needed to understand the underlying mechanisms.

## Data Availability

The raw data supporting the conclusions of this article will be made available by the authors, without undue reservation.
